# Supplementary material for: Cardioprotection Attributed to Aerobic Exercise-Mediated Inhibition of ALCAT1 and Oxidative Stress-Induced Apoptosis in MI Rats
Source: Biomedicines. 2022 Sep 11;10(9):2250. doi: 10.3390/biomedicines10092250 (PMC9496522; doi:10.3390/biomedicines10092250)
Supplement: Supplementary file 1 [file biomedicines-10-02250-s001.zip › biomedicines-1835986-supplementary.pdf]

Supporting Information

Cardioprotection attributed to aerobic exercise-mediated inhibition of ALCAT1 and oxidative stress-induced apoptosis in MI rats

Niu Liu [1,2], Yingni Zhu [1], Wei Song [3], Wujing Ren [3] and Zhenjun Tian [3,*]

[1] School of Physical Education, Weinan Normal University, Weinan 714099 Shaanxi, China
[2] College of P.E and Sports, Beijing Normal University, Beijing 100875, China
[3] Institute of Sports and Exercise Biology, School of Physical Education, Shaanxi Normal University, Xi'an 710119, Shaanxi, China
* Correspondence: tianzhj@snnu.edu.cn

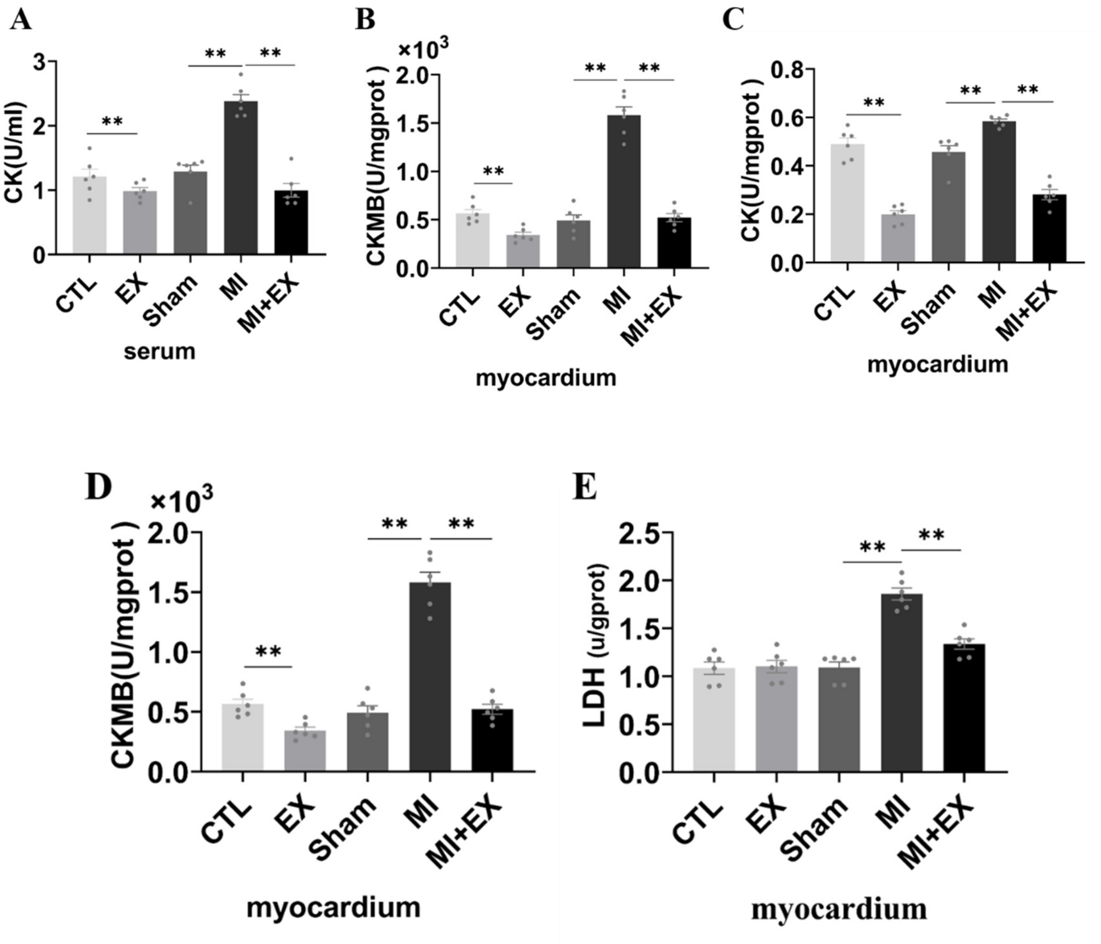

Figure S1. Exercise training reduces myocardial infarct-related enzymes after MI. Elisa kits analysis of myocardial infarct-related enzymes activity, CK **(A, C)**, CKMB **(B, D)** and LDH **(E)** were measured in rat myocardial tissue and serum samples (n=5,6); Values presented are mean ± SEM. CTL, normal control; sham; MI, myocardial infarct; EX, CTL + exercise; MI+EX, MI+ exercise. **P*<0.05, ***P*<0.01.

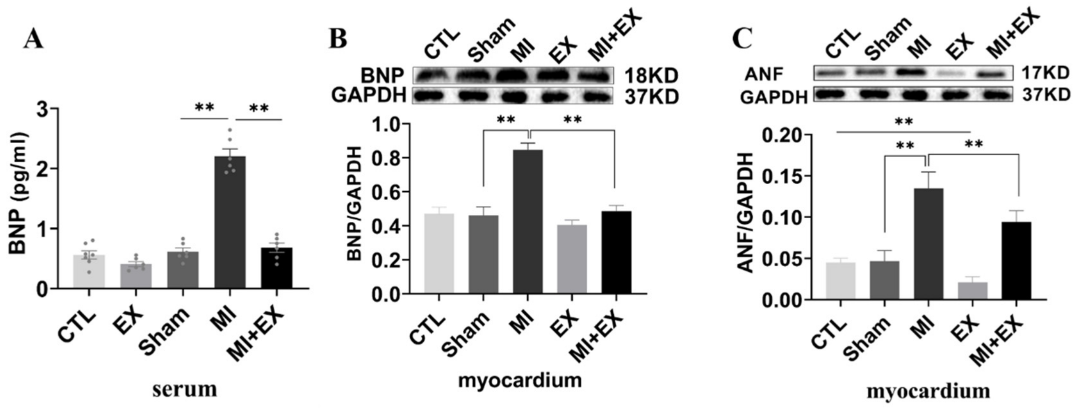

Figure S2. Exercise training mitigates cardiac injury after MI. (A-B) The level of BNP, brain natriuretic peptide, was measured in rat serum and myocardial tissue samples (n=6); **(C)** Western blot images and their densitometric quantitative analysis of ANF (atrial natriuretic factor, n=3). Values presented are mean ± SEM. CTL, normal control; sham; MI, myocardial infarct; EX, CTL + exercise; MI+EX, MI+ exercise.**P*<0.05, ***P*<0.01.
